# Gut microbiome shifts in people with type 1 diabetes are associated with glycaemic control: an INNODIA study

**DOI:** 10.1007/s00125-024-06192-7

**Published:** 2024-06-04

**Authors:** Tommi Vatanen, Carine de Beaufort, M. Loredana Marcovecchio, Lut Overbergh, Soren Brunak, Mark Peakman, Chantal Mathieu, Mikael Knip, on behalf of the INNODIA consortium

**Affiliations:** 1 Research Program for Clinical and Molecular Metabolism, Faculty of Medicine, University of Helsinki, Helsinki, Finland; 2 Institute of Biotechnology, Helsinki Institute of Life Science (HiLIFE), University of Helsinki, Helsinki, Finland; 3 Department of Microbiology, Faculty of Agriculture and Forestry, University of Helsinki, Helsinki, Finland; 4 Broad Institute of MIT and Harvard, Cambridge, MA USA; 5 Liggins Institute, University of Auckland, Auckland, New Zealand; 6 Paediatric Endocrinology and Diabetology (DECCP), Centre Hospitalier de Luxembourg, Luxembourg, Luxembourg; 7 Faculty of Science, Technology and Medicine, University of Luxembourg, Esch-sur-Alzette, Luxembourg; 8 Department of Paediatrics, University of Cambridge, Cambridge, UK; 9 Katholieke Universiteit Leuven/Universitaire Ziekenhuizen, Leuven, Belgium; 10 Novo Nordisk Foundation Center for Protein Research, Faculty of Health and Medical Sciences, University of Copenhagen, Copenhagen, Denmark; 11 Immunology & Inflammation Research Therapeutic Area, Sanofi, Cambridge, MA USA; 12 Department of Chronic Diseases and Metabolism, Endocrinology, Katholieke Universiteit Leuven, Leuven, Belgium; 13 New Children’s Hospital, Helsinki University Hospital, Helsinki, Finland; 14 Tampere Center for Child Health Research, Tampere University Hospital, Tampere, Finland

**Keywords:** C-peptide, *Faecalibacterium prausnitzii*, First-degree relatives, Gut microbiome, HbA_1c_, Newly diagnosed

## Abstract

**Aims/hypothesis:**

The gut microbiome is implicated in the disease process leading to clinical type 1 diabetes, but less is known about potential changes in the gut microbiome after the diagnosis of type 1 diabetes and implications in glucose homeostasis. We aimed to analyse potential associations between the gut microbiome composition and clinical and laboratory data during a 2 year follow-up of people with newly diagnosed type 1 diabetes, recruited to the Innovative approaches to understanding and arresting type 1 diabetes (INNODIA) study. In addition, we analysed the microbiome composition in initially unaffected family members, who progressed to clinical type 1 diabetes during or after their follow-up for 4 years.

**Methods:**

We characterised the gut microbiome composition of 98 individuals with newly diagnosed type 1 diabetes (ND cohort) and 194 autoantibody-positive unaffected family members (UFM cohort), representing a subgroup of the INNODIA Natural History Study, using metagenomic sequencing. Participants from the ND cohort attended study visits within 6 weeks from the diagnosis and 3, 6, 12 and 24 months later for stool sample collection and laboratory tests (HbA_1c_, C-peptide, diabetes-associated autoantibodies). Participants from the UFM cohort were assessed at baseline and 6, 12, 18, 24 and 36 months later.

**Results:**

We observed a longitudinal increase in 21 bacterial species in the ND cohort but not in the UFM cohort. The relative abundance of *Faecalibacterium prausnitzii* was inversely associated with the HbA_1c_ levels at diagnosis (*p*=0.0019). The rate of the subsequent disease progression in the ND cohort, as assessed by change in HbA_1c_, C-peptide levels and insulin dose, was associated with the abundance of several bacterial species. Individuals with rapid decrease in C-peptide levels in the ND cohort had the lowest gut microbiome diversity. Nineteen individuals who were diagnosed with type 1 diabetes in the UFM cohort had increased abundance of *Sutterella* sp. *KLE1602* compared with the undiagnosed UFM individuals (*p*=1.2 × 10^−4^).

**Conclusions/interpretation:**

Our data revealed associations between the gut microbiome composition and the disease progression in individuals with recent-onset type 1 diabetes. Future mechanistic studies as well as animal studies and human trials are needed to further validate the significance and causality of these associations.

**Graphical Abstract:**

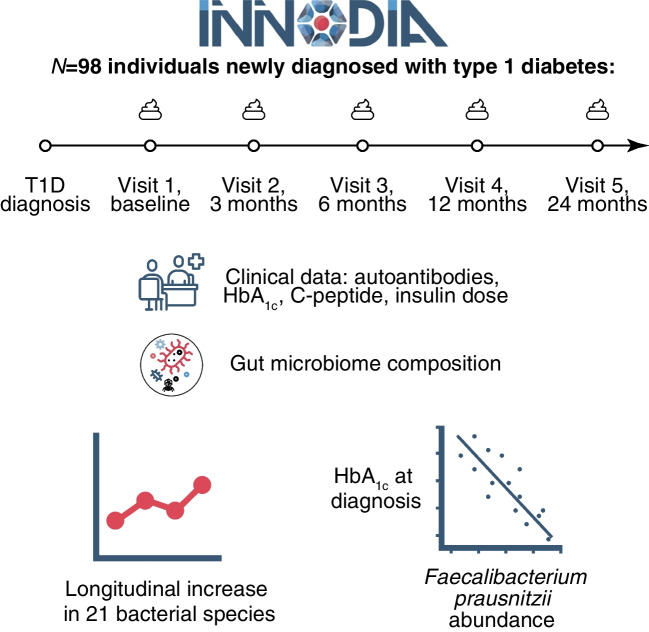

**Supplementary Information:**

The online version of this article (10.1007/s00125-024-06192-7) contains peer-reviewed but unedited supplementary material.


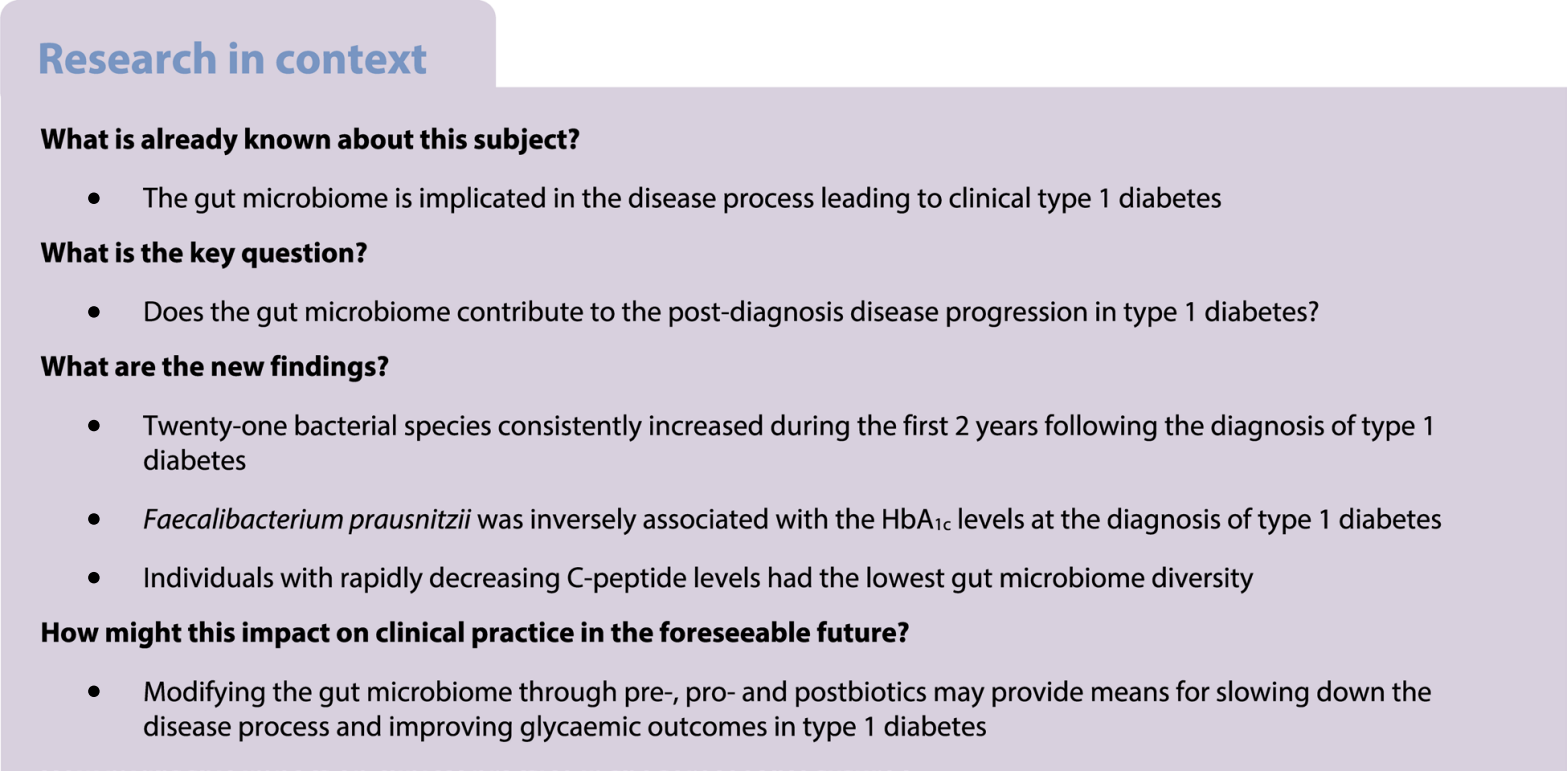


## Introduction

Mounting evidence indicates an intricate relationship between the gut microbiome (GM), a complex consortium of microbes inhabiting the lower gastrointestinal tract, and type 1 diabetes. Both human cohort studies [1–7] and controlled animal experiments [8–12] have demonstrated that the GM harbours both protective and harmful features that may influence the disease process leading to type 1 diabetes. For example, microbially produced short-chain fatty acids (SCFAs) provided protection from type 1 diabetes [1, 7, 12, 13]. Further complicating such analyses, HLA allele combinations providing increased risk for type 1 diabetes also result in changes in the GM composition, potentially through host regulation and selection [14]. Recent and ongoing trials explore the possibility to modify the microbiome-linked disease risk through various interventions targeting the GM [13, 15].

The GM is also implicated in the natural course of type 1 diabetes after the diagnosis [13, 16–18]. For example, existing evidence suggests that the GM is involved in regulating host glycaemic control [13, 19]. An exploratory faecal microbiome transplant (FMT) trial in individuals with recent-onset type 1 diabetes found that autologous FMT halted the decline in endogenous insulin secretion [20]. Together, these emerging data suggest that GM modifications could provide possibilities to intervene in the disease process and even slow down its progression. To this end, a combination of exploratory analyses and controlled experiments is needed to identify microbial strains, metabolites and other GM features that are implicated in host physiology and type 1 diabetes-related biomarkers.

Here, we analysed the GM profiles, for associations with clinical and laboratory data, from people with newly diagnosed type 1 diabetes and unaffected autoantibody (AAB)-positive family members participating in the European, multicentre Innovative approaches to understanding and arresting type 1 diabetes (INNODIA) Natural History Study [21] during a follow-up period of 2–4 years. INNODIA has collected rich information and clinical data on the participants, including fasting C-peptide and HbA_1c_ measurements, to assess endogenous insulin production and glycaemic control, respectively. We report associations between host glycaemic control, diabetes progression and GM features.

## Methods

### Study population

This study recruited two cohorts from the large INNODIA Natural History Study [21]. The first cohort comprised individuals with newly diagnosed type 1 diabetes (ND cohort) [22] and the other cohort AAB-positive unaffected family members of individuals with type 1 diabetes (UFM cohort) (Table 1). Participants were identified through adult and paediatric diabetes clinics at participating sites and recruited between November 2016 and November 2021. The sex distribution among ND participants was quite even, while there was a female preponderance among the UFM participants, which may reflect a higher willingness to participate in the study among female family members, particularly mothers of ND participants. The overwhelming majority of the participants were white. The ND participants are representative of European individuals with newly diagnosed type 1 diabetes. The UFM participants are representative of European family members of ND individuals, except for the uneven sex distribution. We do not have information on the socioeconomic characteristics of the participants.

**Table 1:** Clinical and demographic data of the study participants from the ND and UFM cohorts

Characteristic	ND	UFM
Total participants (*N*)	98	194
Sex		
Female	48 (49)	107 (55)
Male	50 (51)	87 (45)
Age at diagnosis/recruitment		
Mean (SD)	12.30 (8.64)	21.2 (14.1)
Median (IQR) [Q1, Q3]	11 (8) [7, 15]	16 (26) [9, 35]
BMI SDS at baseline		
Mean (SD)	0.41 (1.11)	N/A
Median (IQR) [Q1, Q3]	0.40 (1.61) [−0.40, 1.21]	N/A
Glucose reading at baseline (mmol/mol)		
Mean (SD)	7.73 (4.68)	N/A
Median (IQR) [Q1, Q3]	6.40 (2.77) [5.30, 8.08]	N/A
HbA_1c_ at diagnosis (mmol/mol)		
Mean (SD)	101 (26.9)	N/A
Median (IQR) [Q1, Q3]	104 (35) [87, 122]	N/A
HbA_1c_ at diagnosis (%)		
Mean (SD)	11.4 (4.6)	N/A
Median (IQR) [Q1, Q3]	11.7 (3.2) [10.1, 13.3]	N/A
HbA_1c_ at baseline (mmol/mol)		
Mean (SD)	75.3 (23.2)	33.7 (4.6)
Median (IQR) [Q1, Q3]	78.5 (25.5) [60.8, 86.2]	34 (4.9) [31.1, 36.0]
HbA_1c_ at baseline (%)		
Mean (SD)	9.0 (4.3)	5.3 (2.6)
Median (IQR) [Q1, Q3]	9.3 (2.3) [7.7, 10.0]	5.3 (0.4) [5.0, 5.4]
Insulin dose at baseline (IU/kg)		
Mean (SD)	0.52 (0.26)	N/A
Median (IQR) [Q1, Q3]	0.53 (0.32) [0.35, 0.67]	N/A
Fasted C-peptide at baseline (pmol/l)		
Mean (SD)	272 (233)	N/A
Median (IQR) [Q1, Q3]	211 (236) [106, 342]	N/A
GADA		
Negative	26 (27)	74 (38)
Positive	72 (73)	120 (62)
IA-2A		
Negative	30 (31)	161 (83)
Positive	68 (69)	33 (17)
IA		
Negative	24 (24)	102 (53)
Positive	74 (76)	92 (47)
ZnT8A		
Negative	35 (36)	150 (77)
Positive	63 (64)	44 (23)
Detectable AABs		
Mean (SD)	2.83 (0.93)	1.49 (0.95)
Median (IQR) [min, max]	3 (2) [1, 4]	1 (1) [0, 4]

Data are *n* (%) unless otherwise noted

IAs were measured after the patients had already received exogenous insulin which is known to induce IA production, and these antibodies are not distinguishable from IAAs by the assay used

N/A, not applicable

Q1, first quartile

Q3, third quartile

INNODIA selected the first 100 ND cohort individuals for in-depth molecular assays, including GM profiling. Stool samples were available for 98 ND cohort participants. The UFM cohort was included to assess possible GM shifts during the asymptomatic stage of type 1 disease. UFM participants in this study were selected based on stool sample availability. A minority of the UFM participants (10/194; 5.2%) in this study were related to the ND participants. Both ND and UFM participants tested positive for at least one diabetes-associated AAB out of the three analysed (GADA, IA-2A and ZnT8A). Insulin antibodies (IAs) were measured after the ND participants had already received exogenous insulin which is known to induce IA production, and these antibodies are not distinguishable from IAA by the assay used. BMI was calculated as the weight in kilograms divided by the square of the height in metres. Age- and sex-appropriate standard deviation scores (SDSs) were calculated using World Health Organization 2006 and 2007 data [23]. A harmonised protocol for sample collection and storage was used in the study centres [21]. The study followed the guidelines of the Declaration of Helsinki for research on human participants, and the study protocols were approved by the ethical committees of the participating hospitals. Either the parent or participants themselves gave their written informed consent. Sex of the participants was self-reported. Sex and gender were not inclusion or exclusion criteria in the study.

### Stool sample collection

Stool samples were collected from the INNODIA ND cohort at baseline (within 6 weeks after the diagnosis of type 1 diabetes) and at 3, 6, 12 and 24 months later. In the INNODIA UFM cohort, stool samples were collected at 6, 12, 18, 24 and 36 months after the screening visit (baseline). The samples were collected in OMNIgene-Gut OMR-200 collection tubes (DNA Genotek, Ottawa, ON, Canada), which stabilise DNA at ambient temperature for up to 60 days. Participants were asked to collect the stool samples at home during the week preceding the next study centre visit and to bring the sample with them to the study centre. The samples were frozen at −80°C in the local study centre and shipped frozen to the INNODIA Biobank for storage at −80°C until analysis.

### DNA extraction

DNA was extracted from 250 μl aliquots of the faecal samples in OMNIgene-Gut OMR-200 collection tubes using the NucleoSpin 96 Soil (Macherey-Nagel, Hoerdt, France) kit. Bead beating was done horizontally on a Vortex-Genie 2 (Scientific Industries, NY, USA) at 2700 rev/min for 5 min. One negative control and one positive control (ZymoBIOMICS Microbial Community Standard, Zymo Research, CA, USA) were included per batch of samples from the DNA extraction and throughout the laboratory process.

### DNA sequencing

The quality of extracted DNA was evaluated using agarose gel electrophoresis. The quantity of DNA was measured by a Qubit 2.0 fluorometer (ThermoFisher Scientific, MA, USA). DNA was randomly sheared into fragments of 350 bp, on average. Sequencing libraries were constructed using NEBNext Ultra Library Prop Kit (New England Biolabs, Herts, UK). The quality of the DNA libraries was measured using a Qubit 2.0 fluorometer and Agilent 2100 Bioanalyzer (Agilent Technologies, CA, USA), to assess the fragment size distribution. The DNA concentration of the final library was determined by quantitative real-time PCR (qPCR) prior to sequencing. Metagenomic DNA was sequenced using a 2 × 150 bp paired-end protocol on an Illumina platform (Illumina, CA, USA).

### Sequence data quality control

Quality control of sequencing reads was conducted using KneadData (v.0.6.1; https://huttenhower.sph.harvard.edu/kneaddata/) to remove low-quality reads and trim low-quality bases. Trimmed reads shorter than 100 bases and reads mapping to human genome GRCh38 were discarded.

### Reference gene catalogue

The Clinical Microbiomics Human Gut HG04 gene catalogue, consisting of 14,355,839 genes, was used as a reference gene catalogue. The catalogue is based on 12,170 non-public human gut samples, 9428 publicly available metagenomes from 43 countries [24] and 3567 publicly available genome assemblies from isolated microbial strains. Taxonomic abundance profiles were obtained using the Clinical Microbiomics HGMGS version HG4.D.1 set of 2095 metagenomic species (MGS), each represented by a set of genes with highly coherent abundance profiles and base compositions in the 12,170 metagenomes. The MGS concept is described in [25]. Quality controlled reads were mapped to the gene catalogue using the BWA mem (v.0.7.16a; https://bio-bwa.sourceforge.net/) algorithm [26] with the following criteria: mapping quality (MAPQ) ≥20, sequence identity ≥95% over ≥100 bp.

### MGS annotations

An MGS was taxonomically annotated by a BLAST search (https://blast.ncbi.nlm.nih.gov/Blast.cgi) of its genes against the NCBI RefSeq genome database (27 January 2020) combined with rank-specific annotation criteria. An MGS was assigned to a taxon if at least M% of its genes were mapped to the taxon and no more than D% of its genes were mapped to a different taxon. We considered only blast hits with an alignment length ≥100 bp, ≥50% query coverage and percentage identity ≥PID. Parameters M, D and PID were defined for subspecies, species, genus, family, order, class, phylum and superkingdom as follows: PID = (95, 95, 85, 75, 65, 55, 50, 45); M = (75, 75, 60, 50, 40, 30, 25, 20); and D = (10, 10, 10, 20, 20, 20, 20, 15), respectively. Finally, each MGS was processed with CheckM (v.1.1.2; https://ecogenomics.github.io/CheckM/) [27], and the annotation was updated with the CheckM result if this resulted in a lower taxonomic rank.

### MGS abundance calculation

MGS abundances were calculated as described in [25]. Briefly, an abundance for each MGS was estimated using gene abundances of 100 signature genes optimised for accurate abundance profiling. MGS abundances were normalised to account for length of the signature genes and the relative abundance of the data.

### Functional annotation and profiling

We measured microbiome functional modules using the gut metabolic modules (GMMs) which consist of 103 conserved metabolic pathways, each defined as a series of enzymatic steps represented by KEGG Orthology identifiers (KOs) [28]. Abundance of a functional module was calculated as the proportion of all mapped reads that mapped to a KO belonging to the given module.

### Diversity estimation

The α and β diversities were estimated using rarefied MGS abundances. We used the number of entities detected (richness) and Shannon’s index to measure α diversity. The β diversity was calculated using the Bray–Curtis dissimilarity.

### Statistical analysis

Associations between microbial α diversities and clinical covariates were tested using linear models. Associations between microbial β diversities and clinical covariates were tested with permutational multivariate analysis of variance (PERMANOVA) as implemented in vegan R package. Associations between individual microbial features (species, functional modules) were tested by linear mixed effect models in MaAsLin2 R package [29], assuming normally distributed data and normally distributed random intercepts per study participant. The statistical models included covariates to correct for effects related to the clinical site, sex and age at diagnosis (age at recruitment for the UFM cohort). Statistical models involving longitudinal analyses additionally included covariates to correct for the time from baseline and participant-specific random intercepts. All data analyses were conducted in R v.4.0.0 [30] in the INNODIA Cloud environment. False discovery rate (FDR) correction of all tests including multiple features was performed using the Benjamini–Hochberg procedure and the resulting *q* values (i.e. FDR-corrected *p* values) are reported when appropriate.

## Results

**Fig. 1:**
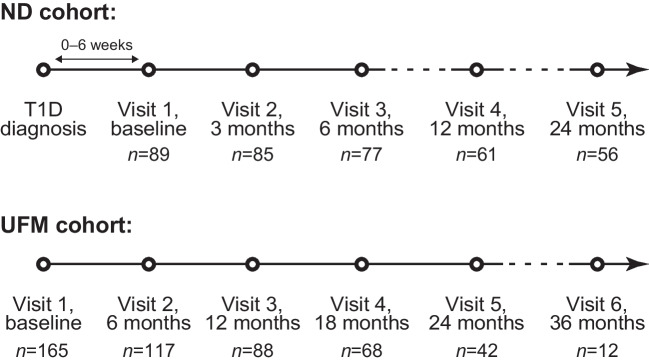
Stool sample collection and metagenomes in the ND (*N*=98 individuals) and UFM (*N*=194 individuals) cohorts within the INNODIA study. Stool samples were collected at home during the week preceding the next study centre visit. *n* shows the number of metagenomes generated per time point. T1D, type 1 diabetes

We analysed 368 faecal samples collected from 98 individuals newly diagnosed with type 1 diabetes (ND cohort) and 492 faecal samples collected from 194 UFM participants (Fig. 1, Table 1). Participants were recruited across 25 clinical centres in 13 European countries (Table 2). The age of the ND participants varied from 1 to 38 years (mean 12.3, SD 8.64) and the cohort comprised 48 female and 50 male study participants. The average age at the diagnosis was 12.3 years (SD 8.6) and the mean diabetes duration was 3.7 weeks (SD 1.6, minimum 0.7, maximum 6.3 weeks) at the first study visit. At baseline, the average total daily insulin dose was 0.52 IU/kg (SD 0.26), HbA_1c_ 75.3 mmol/mol (SD 23.2; 9.0% [4.0%]) and the mean fasting C-peptide level 272 pmol/l (IQR 236 [Q1, Q3: 106, 342]). The mean plasma glucose reading at baseline was 7.73 mmol/l (IQR 2.77 [Q1, Q3: 5.30, 8.08]). The mean BMI SDS was 0.41 (SD 1.11). Following the results of a previous INNODIA study, we divided the ND cohort participants into three groups based on the change of C-peptide levels over time, depicting the rate of disease progression: rapid decline, slow decline and increasing [31]. The age of the 194 UFM participants varied from 1 to 44 years (mean 21.2, SD 14.1). Clinical and demographic data of both cohorts are presented in Table 1. Stool samples were metagenomically sequenced with an average depth of 9 Gb per sample, corresponding to 30.0 million read pairs per sample (Illumina 2 × 150 paired-end). On average, 87.6% of the high-quality microbiome reads from a sample were mapped to the gut gene catalogue.

**Table 2:** Number of participants (*N*) per clinical centre and study cohort

Clinical centre	Country	*N* (ND)	*N* (UFM)
UH – University of Helsinki	Finland	29	22
ULI – University of Ljubljana	Slovenia	18	7
HH-RH – Herlev University Hospital	Denmark	17	6
UCAM – University of Cambridge	UK	8	10
MUG – Medical University of Graz	Austria	5	11
SUM – Slaski Uniwersytet Medyczny w Katowicach	Poland	4	9
ULB – Universite Libre de Bruxelles	Belgium	3	19
UOUL – Oulun Yliopisto	Finland	2	13
HKA – Hannoversche Kinderheilanstalt	Germany	2	12
CHL – Centre Hospitalier de Luxembourg	Luxembourg	2	9
UNISI – Universita degli Studi di Siena	Italy	2	6
UK – Norfolk and Norwich	UK	2	5
UK – Barts Health NHS Trust	UK	2	
KU Leuven	Belgium	1	21
UULM – Universitat Ulm	Germany	1	1
OUS – Oslo Universitetssykehus HF	Norway		13
Paris – Hospital Robert Debre	France		8
Lund University	Sweden		5
Inserm – Institut National de la Sante et de la Recherche Medicale	France		4
Paris – Centre Hospitalier Sud-Francilien	France		3
B – ZH Geel	Belgium		2
Paris – Hospital Jean-Verdier	France		2
UK – Northampton General Hospital	UK		2
UK – Birmingham Children's Hospital	UK		2
IT – Ospedale Paediatrico Bambino Gesu	Italy		1
MUW – Medical University Vienna	Austria		1

**Fig. 2:**
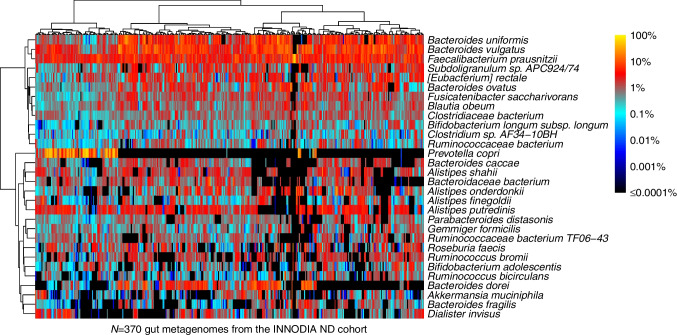
Heatmap displaying relative abundances of the 30 most abundant bacterial species in *N*=370 GM profiles from the participants in the INNODIA ND cohort. Row and column orders were determined by hierarchical clustering using Ward’s clustering criterion

We first analysed the metagenomes from the ND cohort to investigate links between disease biomarkers and the GM following the diagnosis of type 1 diabetes. The most prevalent and abundant GM species included several *Bifidobacterium* and *Bacteroides* species as well as *Faecalibacterium prausnitzii*, all common human GM members (Fig. 2). We observed a stark dichotomy in the relative abundance of *Prevotella copri*, which was highly abundant in a subset of the metagenomes (*n*=88, 23.8%) and missing in others. *P.* *copri* presence was associated with change in abundance of 42 microbiome functional modules (Wilcoxon test, FDR-corrected *p*<0.05, electronic supplementary material [ESM] Table 1).

We tested for associations between microbial α and β diversities and demographic/clinical/biochemical data at baseline in the ND cohort. We detected shifts in microbial profiles (β diversities) between the clinical centres (PERMANOVA test, *R*^2^=0.183, *p*=0.007). There were no detectable shifts or associations between microbial α or β diversities and participants’ sex, age, BMI SDS, insulin dose, HbA_1c_, fasting C-peptide measurement or C-peptide/glucose ratio at baseline (α diversities, linear mixed model, *p*>0.1; β diversities, PERMANOVA test, *p*>0.05). We observed associations between the abundance of 60 bacterial species and the clinical centres (*q*<0.20, ESM Table 2).

**Fig. 3:**
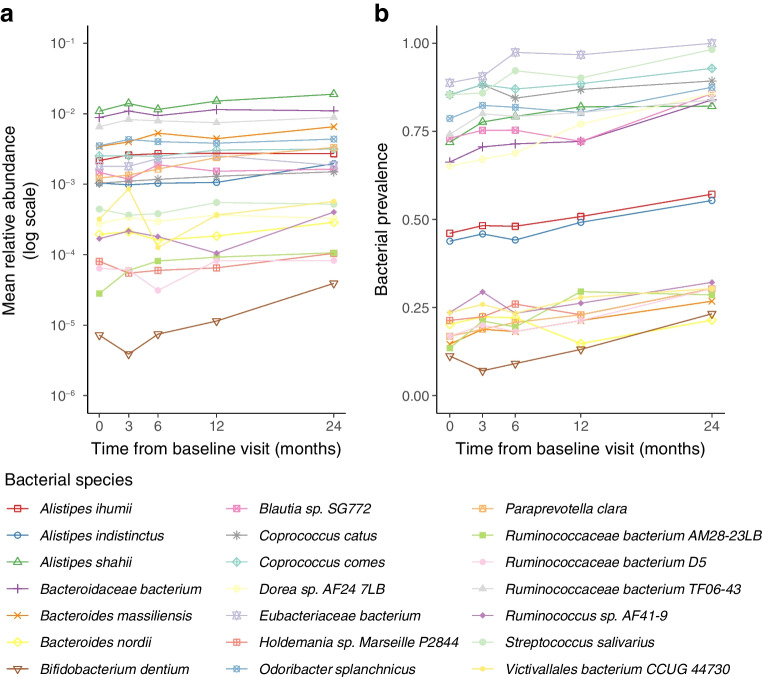
Longitudinal changes in the ND cohort during the follow-up. (**a**) Relative abundance and (**b**) prevalence of *n*=21 bacterial species had statistically significant longitudinal trends (linear mixed effects model, *q*<0.10)

We next pooled data from the follow-up period to assess longitudinal microbiome changes over the visit schedule in the ND cohort. Twenty-one microbial species and eight functional modules showed longitudinal trends (linear mixed effects model, *q*<0.20, Fig. 3a,b, ESM Table 2). All these species had an increasing trend over time, and they included both common and rare species with high and low average relative abundances.

**Fig. 4:**
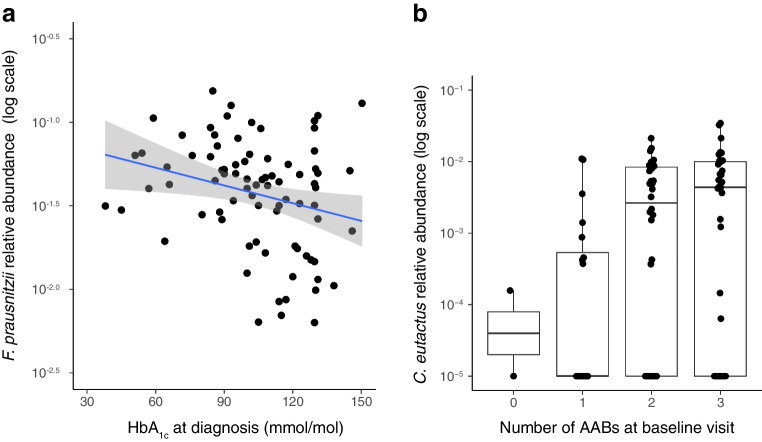
Associations between gut bacteria at baseline and clinical covariates in the ND cohort. (**a**) Relative abundance of *F. prausnitzii* was inversely associated with HbA_1c_ values at the diagnosis of type 1 diabetes (linear model, *q*=0.19). (**b**) Relative abundance of *C. eutactus* correlated with the number of AABs at the baseline visit (linear model, *q*=0.16)

We tested for association between HbA_1c_ values at the time of the diagnosis of type 1 diabetes and the microbiome at the baseline visit (within 6 weeks after the diagnosis) in the ND cohort. The relative abundance of *F. prausnitzii* was inversely correlated with HbA_1c_ (linear mixed effects model, β-coefficient=−0.15 [95% CI −0.24, −0.058], *q*=0.19, nominal *p*=0.0019, Fig. 4a, ESM Table 3) and microbial hydrogen metabolism (functional module MF0098) was positively associated with HbA_1c_ (β-coefficient=2.43 [95% CI 0.92, 3.94], *q*=0.17, nominal *p*=0.0020, ESM Table 3). Since participants with ketoacidosis at the diagnosis of type 1 diabetes had significantly higher HbA_1c_ (*p*=5.2 × 10^−5^; the average HbA_1c_ in participants with ketoacidosis was 117.0 mmol/mol [12.9%], and in participants with no ketoacidosis 93.3 mmol/mol [10.7%]), we conducted sensitivity analysis controlling for ketoacidosis. Both associations above became less statistically significant (*F. prausnitzii*, *q*=0.29, nominal *p*=0.0031; hydrogen metabolism, *q*=0.35, nominal *p*=0.0077), indicating that ketoacidosis explained away a fraction of the associations. Ketoacidosis status alone was not associated with any GM features at baseline in the ND cohort.

**Fig. 5:**
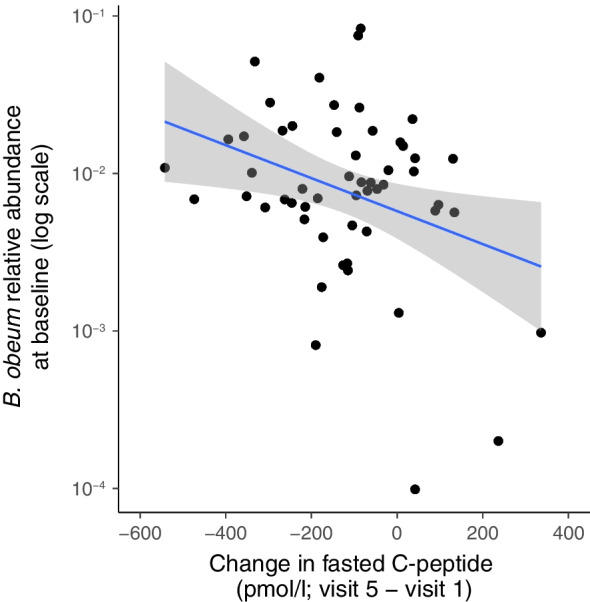
Relative abundance of *B. obeum* was inversely associated with the change in fasting C-peptide concentration during the first 2 years of clinical type 1 diabetes (linear model, *q*=0.07) in the ND cohort

We analysed possible associations of the clinical and laboratory data at the baseline visit (within 6 weeks from the diagnosis) with the baseline microbiome composition in the ND cohort. Tested variables included HbA_1c_ value, insulin dose per kg, fasting C-peptide concentration, fasting C-peptide/glucose ratio and BMI SDS. We did not observe any associations between baseline measurements and microbiome features. We also tested for associations between the number of detected AABs at baseline and the baseline microbiome composition. IAs were excluded from the AAB count since the participants had already received exogenous insulin which is known to induce IA production, and these antibodies are not distinguishable from IAAs by the assay applied. We observed a positive correlation between *Coprococcus eutactus* and the number of detectable AABs at the baseline visit (β-coefficient=0.41 [95% CI 0.16, 0.66], *q*=0.16, nominal *p*=0.0014, Fig. 4b, ESM Table 4) and an inverse correlation between microbial tyrosine degradation and the number of AABs (β-coefficient=−0.48 [95% CI −0.77, −0.19], *q*=0.17, nominal *p*=0.0014, ESM Table 4). In addition, we analysed the data for associations between the change in clinical parameters (HbA_1c_, insulin dose, fasting C-peptide) during the 2 year follow-up and the baseline microbiome composition. Relative abundance of *Blautia obeum* was inversely associated with change in fasting C-peptide between baseline and visit 5 (β-coefficient=−0.25 [95% CI −0.38, −0.12], *q*=0.07, Fig. 5, ESM Table 5).

**Fig. 6:**
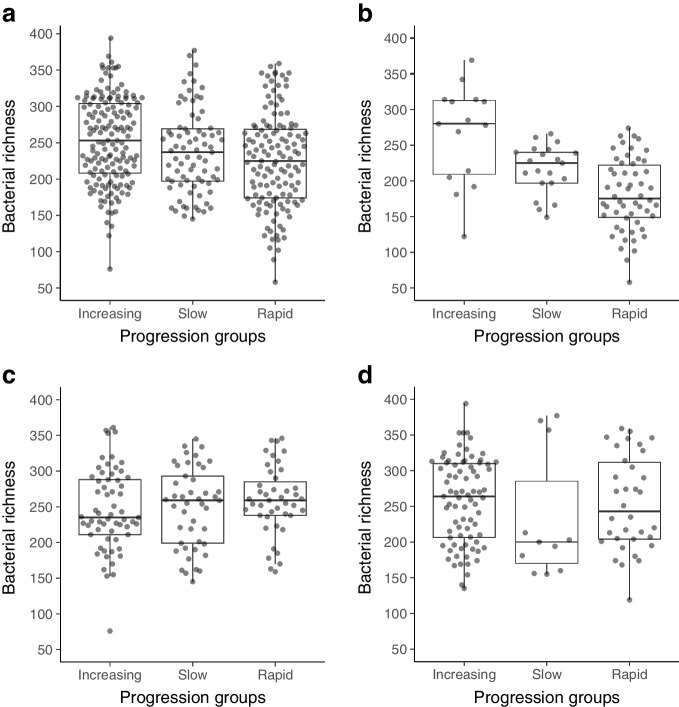
Difference in microbial richness (denoting number of observed species) according to different C-peptide profiles during the follow-up: (**a**) in all ND cohort participants and in three age groups, (**b**) under 7, (**c**) 7–12 and (**d**) over 12 years old, within the ND cohort. Individuals were divided into three groups based on the decline of C-peptide levels over time, depicting the rate of disease progression: rapid and slow progression and increasing C-peptide levels [30] Species richness denotes the number of observed species

We compared the microbiome composition between the three groups of participants in the ND cohort based on the change of C-peptide levels over time and observed differences in *Streptococcus salivarius* (*q*=0.12), *Campylobacter concisus* (*q*=0.15) and *Veillonella atypica* (*q*=0.19) abundances between the groups (ESM Table 6). Bacterial α diversity, measured by richness (number of observed species), differed between the groups (repeated measures analysis of variance, corrected for clinical centres, age groups and an interaction between the age groups and progression groups, *p*=0.021) such that the individuals with rapid C-peptide decline had the lowest bacterial richness, on average (Fig. 6a). As there was a wide age range (1–38 years) within the ND cohort potentially affecting the GM composition, we further divided the participants into three age categories, age <7, age 7–12 and age 13 or older, based on distinct immunohistological profiles [32] and clinical characteristics [33]. Baseline clinical characteristics per age category are shown in ESM Table 7. We found that the association with C-peptide decline was most apparent in the group who were diagnosed with type 1 diabetes before the age of 7 (Fig. 6b–d), although the interaction term between the age groups and the progression groups was not significant (*p*=0.77).

Nineteen individuals in the UFM cohort progressed to clinical type 1 diabetes during or after the follow-up. We compared the GMs of these individuals with the rest of the UFM cohort. Individuals diagnosed with diabetes had increased abundance of *Sutterella* sp. *KLE1602* (longitudinal analysis, β-coefficient=1.20 [95% CI 0.59, 1.80], *q*=0.033, nominal *p*=1.2 × 10^−4^, ESM Table 8), which was also more prevalent in these individuals: 10 out of 19 participants (53%) diagnosed with type 1 diabetes had *Sutterella* sp. *KLE1602* in at least one stool sample compared with 27% (48 of 175) in non-diabetic UFM cohort participants. The progressors also had increased abundance of the functional module *pyruvate:ferredoxin oxidoreductase* (MF0073, β-coefficient=0.018 [95% CI 0.0092, 0.028], *q*=0.059, nominal *p*=9.8 × 10^−5^, ESM Table 8) in their GMs. We did not observe any longitudinal GM changes or associations between HbA_1c_ levels and GM features, or associations between the stages of type 1 diabetes and microbial α diversities in the UFM cohort.

## Discussion

Baseline relative abundance of *F. prausnitzii* was inversely correlated with HbA_1c_ at the diagnosis of type 1 diabetes. *F. prausnitzii* is a beneficial commensal bacterium with important health-promoting functions [34], by, for example, production of butyrate which serves as an energy source for colonocytes. Microbial butyrate production is among the strongest mechanisms through which the GM mediates protection against islet autoimmunity and type 1 diabetes [7, 12, 35]. In a pilot trial supplementing resistant starch modified with SCFAs to humans with type 1 diabetes, individuals with the highest SCFA concentrations exhibited the best glycaemic control [13]. Future human cohort studies with faecal and/or serum butyrate quantification could further investigate the role of butyrate in glycaemic control in type 1 diabetes.

We observed differences in microbial richness, the number of observed taxa, according to the disease progression in the ND cohort. Individuals with the fastest decline in C-peptide levels, reflecting decline in endogenous insulin production‚ had the lowest GM richness. We also observed an inverse association between *B. obeum* and the change in fasting C-peptide levels between baseline and 2 years after the diagnosis. The difference in microbial richness is compatible with the observations from the DIABIMMUNE cohort, which was a longitudinal study in infants and young children with HLA-conferred susceptibility to type 1 diabetes [6]. In that study the decline in microbial diversity appeared prior to the diagnosis of type 1 diabetes. However, such a pattern had been absent in most other human cohort studies, suggesting that microbial diversity alone has a poor predictive value for type 1 diabetes. Together, these observations paint a picture where a multitude of microbial features may be weakly involved in the host–GM interplay in established disease.

UFM cohort members who had been diagnosed with type 1 diabetes during or after the follow-up had higher relative abundance and prevalence of *Sutterella* sp. *KLE1602* compared with other UFM cohort members. While *Sutterella* spp. are not as extensively studied as many other prevalent gut bacteria, a study in mice found that *Sutterella* spp. degraded IgA in the gut [36]. The authors showed that mice with *Sutterella* spp. and resulting low gut IgA levels had increased dextran sulfate sodium (DSS)-induced colon ulceration compared with mice with normal IgA levels and no *Sutterella*. A randomised double-blind placebo-controlled clinical trial in humans testing the efficacy of FMT in ulcerative colitis found that *Sutterella* spp. were enriched in individuals who failed to stay in remission following the transplant [37]. Speculatively, *Sutterella* could disrupt the intestinal antibacterial immune response and indirectly induce local and systemic inflammation by increase in intracellular pathogens and pathobionts [38]. Potential roles of *Sutterella* spp. and colonic IgA in type 1 diabetes could be further investigated by, for example, including faecal IgA assays and sorting strategies that identify gut bacterial IgA coating in current and upcoming type 1 diabetes cohort studies.

We observed longitudinal GM changes over the 2 year observation period in the ND cohort. These changes included increases in known oral species *Bifidobacterium dentium* and *S. salivarius*, as well as several butyrate-producing *Ruminococcaceae* species and *Coprococcus catus*. Such longitudinal shifts were absent in the UFM cohort, suggesting the changes in the ND cohort could be related to the manifestation of type 1 diabetes and subsequent lifestyle changes. For example, individuals with newly diagnosed type 1 diabetes are recommended to adapt their dietary patterns and content of their diet which might be reflected in the GM structure.

We found *P. copri* in 88 (23.8%) metagenomes. Presence of *P. copri* tended to have significant effects in many functional modules encoded by the entire microbial community. *Prevotella*-rich microbiomes are more common in non-Western populations, while the GMs in Western countries are more commonly dominated by *Bacteroides* spp. [39]. Generally, high abundance of *Prevotella* is associated with a plant-rich, high-fibre diet, and overweight adults consuming a whole-grain diet for 6 weeks lost more weight if their GM harboured *Prevotella* [40]. Here, the observed dichotomy in *Prevotella* abundance could reflect long-term dietary habits and other external factors affecting the GM composition. While we did not observe any associations with *P. copri* (or other *Prevotella* spp.) and clinical data in our analyses, *Prevotella*-rich microbiomes could still provide means for stratifying the metagenomes to different community types in further exploratory analyses.

These data included prominent differences in the GM composition between recruiting clinical sites, reflected in β diversities and individual microbial species and functional modules. Such shifts are likely related to geographic, lifestyle, ethnic and other differences between the clinical sites distributed across 13 European countries. These factors are known confounders in microbiome studies since they often prominently correlate with GM structure and therefore pose a challenge to designing large GM studies in rare or otherwise difficult-to-study human conditions. Nonetheless, these data further underscore the necessity of controlling for these and other known confounding factors in GM studies. Specifically, these factors should be considered in trial design for best reproducibility and reliability. Sex was included as a covariate in the statistical analysis, and accordingly sex is not confounding the study results.

### Strengths

This study investigated the GM of a relatively large cohort of 98 individuals with newly diagnosed type 1 diabetes with a 2 year longitudinal follow-up involving five visits to the clinic. On all clinic visits, harmonised clinical data on the participants, including fasting C-peptide and HbA_1c_ measurements, were collected. Additionally, stool samples were collected using a harmonised process, enabling robust comparisons between the study centres.

### Limitations

The GM data included prominent differences between the clinical sites distributed across 13 European countries. Such shifts are likely related to lifestyle, dietary, ethnic and other differences between the populations but since this information has not been collected in INNODIA we were unable to further dissect the source of the observed variation. Among these, the diet is a major GM modulator and dietary changes following type 1 diabetes could plausibly induce shifts in the GM composition. This hypothesis cannot be investigated here due to the lack of information on participant diets. Stool samples and microbiome data were not available at all time points from all individuals. Even though we are not aware of any systematic stool sampling bias, in theory, any such systematic bias could skew the results. Stimulated peak C-peptide level and area under the curve from mixed meal tolerance test (MMTT) are more accurate measures of beta cell function compared with fasting C-peptide used here, but stimulated C-peptide data were not available at baseline for all participants.

### Conclusions

This study reports associations between the GM composition and type 1 diabetes in an INNODIA multicentre human cohort study. Albeit these associations were statistically weak, potential mechanisms behind these associations could be further investigated using animal models and immunological assays. Rigorous, controlled trials are needed to assess the roles of identified bacteria and their potential causality in type 1 diabetes.

## Supplementary Information

Below is the link to the electronic supplementary material.

ESM (PDF 74 KB)

ESM Tables (XLSX 81 KB)

## Data Availability

The data generated and analysed are person-sensitive and can be accessed in secure environments only. Access can be provided by application to the INNODIA Data Access Committee.

## Abbreviations

AAB: Autoantibody
FDR: False discovery rate
FMT: Faecal microbiome transplant
GM: Gut microbiome
IA: Insulin antibody
INNODIA: Innovative approaches to understanding and arresting type 1 diabetes
KO: KEGG Orthology identifier
MGS: Metagenomic species
ND: Newly diagnosed type 1 diabetes participants
PERMANOVA: Permutational multivariate analysis of variance
SCFA: Short-chain fatty acid
SDS: Standard deviation score
UFM: Unaffected family member

## References

[1] de Goffau MC, Luopajärvi K, Knip M et al (2013) Fecal microbiota composition differs between children with β-cell autoimmunity and those without. Diabetes 62(4):1238–1244. 10.2337/db12-0526 23274889 10.2337/db12-0526 PMC3609581

[2] Needell JC, Zipris D (2016) The role of the intestinal microbiome in type 1 diabetes pathogenesis. Curr Diab Rep 16(10):89. 10.1007/s11892-016-0781-z 27523648 10.1007/s11892-016-0781-z

[3] Davis-Richardson AG, Ardissone AN, Dias R et al (2014) Bacteroides dorei dominates gut microbiome prior to autoimmunity in Finnish children at high risk for type 1 diabetes. Front Microbiol 5:678. 10.3389/fmicb.2014.00678 25540641 10.3389/fmicb.2014.00678 PMC4261809

[4] Endesfelder D, zu Castell W, Ardissone A et al (2014) Compromised gut microbiota networks in children with anti-islet cell autoimmunity. Diabetes 63(6):2006–2014. 10.2337/db13-1676 24608442 10.2337/db13-1676

[5] Maffeis C, Martina A, Corradi M et al (2016) Association between intestinal permeability and fecal microbiota composition in Italian children with beta cell autoimmunity at risk for type 1 diabetes. Diabetes Metab Res Rev 32:700–709. 10.1002/dmrr.2790 26891226 10.1002/dmrr.2790

[6] Kostic AD, Gevers D, Siljander H et al (2015) The dynamics of the human infant gut microbiome in development and in progression toward type 1 diabetes. Cell Host Microbe 17(2):260–273. 10.1016/j.chom.2015.01.001 25662751 10.1016/j.chom.2015.01.001 PMC4689191

[7] Vatanen T, Franzosa EA, Schwager R et al (2018) The human gut microbiome in early-onset type 1 diabetes from the TEDDY study. Nature 562(7728):589–594. 10.1038/s41586-018-0620-2 30356183 10.1038/s41586-018-0620-2 PMC6296767

[8] Wen L, Ley RE, Volchkov PY et al (2008) Innate immunity and intestinal microbiota in the development of Type 1 diabetes. Nature 455(7216):1109–1113. 10.1038/nature07336 18806780 10.1038/nature07336 PMC2574766

[9] Burrows MP, Volchkov P, Kobayashi KS, Chervonsky AV (2015) Microbiota regulates type 1 diabetes through Toll-like receptors. Proc Natl Acad Sci U S A 112(32):9973–9977. 10.1073/pnas.1508740112 26216961 10.1073/pnas.1508740112 PMC4538618

[10] Girdhar K, Huang Q, Chow I-T et al (2022) A gut microbial peptide and molecular mimicry in the pathogenesis of type 1 diabetes. Proc Natl Acad Sci U S A 119(31):e2120028119. 10.1073/pnas.2120028119 35878027 10.1073/pnas.2120028119 PMC9351354

[11] Lv W, Graves DT, He L et al (2020) Depletion of the diabetic gut microbiota resistance enhances stem cells therapy in type 1 diabetes mellitus. Theranostics 10(14):6500–6516. 10.7150/thno.44113 32483466 10.7150/thno.44113 PMC7255019

[12] Mariño E, Richards JL, McLeod KH et al (2017) Gut microbial metabolites limit the frequency of autoimmune T cells and protect against type 1 diabetes. Nat Immunol 18:552–562. 10.1038/ni.3713 28346408 10.1038/ni.3713

[13] Bell KJ, Saad S, Tillett BJ et al (2022) Metabolite-based dietary supplementation in human type 1 diabetes is associated with microbiota and immune modulation. Microbiome 10(1):9. 10.1186/s40168-021-01193-9 35045871 10.1186/s40168-021-01193-9 PMC8772108

[14] Russell JT, Roesch LFW, Ördberg M et al (2019) Genetic risk for autoimmunity is associated with distinct changes in the human gut microbiome. Nat Commun 10(1):3621. 10.1038/s41467-019-11460-x 31399563 10.1038/s41467-019-11460-x PMC6689114

[15] Ziegler A-G, Arnolds S, Kölln A et al (2021) Supplementation with Bifidobacterium longum subspecies infantis EVC001 for mitigation of type 1 diabetes autoimmunity: the GPPAD-SINT1A randomised controlled trial protocol. BMJ Open 11(11):e052449. 10.1136/bmjopen-2021-052449 34753762 10.1136/bmjopen-2021-052449 PMC8578987

[16] Yuan X, Wang R, Han B et al (2022) Functional and metabolic alterations of gut microbiota in children with new-onset type 1 diabetes. Nat Commun 13(1):6356. 10.1038/s41467-022-33656-4 36289225 10.1038/s41467-022-33656-4 PMC9606127

[17] Luo S, Yue T, Liu Z et al (2022) Gut microbiome and metabolic activity in type 1 diabetes: an analysis based on the presence of GADA. Front Endocrinol 13:938358. 10.3389/fendo.2022.938358 10.3389/fendo.2022.938358 PMC9563112 36246882

[18] Mejía-León ME, Petrosino JF, Ajami NJ, Domínguez-Bello MG, de la Barca AMC (2014) Fecal microbiota imbalance in Mexican children with type 1 diabetes. Sci Rep 4(1):3814. 10.1038/srep03814 24448554 10.1038/srep03814 PMC3898044

[19] Shilo S, Godneva A, Rachmiel M et al (2022) The gut microbiome of adults with type 1 diabetes and Its association with the host glycemic control. Diabetes Care 45:555–563. 10.2337/dc21-1656 35045174 10.2337/dc21-1656 PMC13053884

[20] de Groot P, Nikolic T, Pellegrini S et al (2020) Faecal microbiota transplantation halts progression of human new-onset type 1 diabetes in a randomised controlled trial. Gut 70:92–105. 10.1136/gutjnl-2020-322630 33106354 10.1136/gutjnl-2020-322630 PMC7788262

[21] Dunger DB, Bruggraber SFA, Mander AP et al (2022) INNODIA Master Protocol for the evaluation of investigational medicinal products in children, adolescents and adults with newly diagnosed type 1 diabetes. Trials 23(1):414. 10.1186/s13063-022-06259-z 35585600 10.1186/s13063-022-06259-z PMC9116021

[22] Marcovecchio ML, Hendriks AEJ, Delfin C et al (2024) The INNODIA Type 1 Diabetes Natural History Study – a European cohort of newly diagnosed children, adolescents and adults. Diabetologia 67:995–1008. 10.1007/s00125-024-06124-5 38517484 10.1007/s00125-024-06124-5 PMC11058619

[23] Cole TJ (1990) The LMS method for constructing normalized growth standards. Eur J Clin Nutr 44(1):45–60 2354692

[24] Pasolli E, Asnicar F, Manara S et al (2019) Extensive unexplored human microbiome diversity revealed by over 150,000 genomes from metagenomes spanning age, geography, and lifestyle. Cell 176(3):649-662.e20. 10.1016/j.cell.2019.01.001 30661755 10.1016/j.cell.2019.01.001 PMC6349461

[25] Nielsen HB, Almeida M, Juncker AS et al (2014) Identification and assembly of genomes and genetic elements in complex metagenomic samples without using reference genomes. Nat Biotechnol 32(8):822–828. 10.1038/nbt.2939 24997787 10.1038/nbt.2939

[26] Li H, Durbin R (2009) Fast and accurate short read alignment with Burrows-Wheeler transform. Bioinformatics 25(14):1754–1760. 10.1093/bioinformatics/btp324 19451168 10.1093/bioinformatics/btp324 PMC2705234

[27] Parks DH, Imelfort M, Skennerton CT, Hugenholtz P, Tyson GW (2015) CheckM: assessing the quality of microbial genomes recovered from isolates, single cells, and metagenomes. Genome Res 25(7):1043–1055. 10.1101/gr.186072.114 25977477 10.1101/gr.186072.114 PMC4484387

[28] Vieira-Silva S, Falony G, Darzi Y et al (2016) Species-function relationships shape ecological properties of the human gut microbiome. Nat Microbiol 1(8):16088. 10.1038/nmicrobiol.2016.88 27573110 10.1038/nmicrobiol.2016.88

[29] Mallick H, Rahnavard A, McIver LJ et al (2021) Multivariable association discovery in population-scale meta-omics studies. PLoS Comput Biol 17(11):e1009442. 10.1371/journal.pcbi.1009442 34784344 10.1371/journal.pcbi.1009442 PMC8714082

[30] R Core Team (2013) R: A language and environment for statistical computing. R Foundation for Statistical Computing, Vienna, Austria

[31] Armenteros JJA, Brorsson C, Johansen CH et al (2023) Multi-omics analysis reveals drivers of loss of β-cell function after newly diagnosed autoimmune type 1 diabetes: An INNODIA multicenter study. MedRxiv 2023.03.22.23287261 (Preprint). 28 March 2023. Available from: https://www.medrxiv.org/content/10.1101/2023.03.22.23287261v1 10.1002/dmrr.3833 38961656

[32] Arif S, Leete P, Nguyen V et al (2014) Blood and islet phenotypes indicate immunological heterogeneity in type 1 diabetes. Diabetes 63(11):3835–3845. 10.2337/db14-0365 24939426 10.2337/db14-0365 PMC4207393

[33] Parviainen A, Härkönen T, Ilonen J, But A, Knip M, Finnish Pediatric Diabetes Register (2022) Heterogeneity of type 1 diabetes at diagnosis supports existence of age-related endotypes. Diabetes Care 45(4):871–879. 10.2337/dc21-1251 35147706 10.2337/dc21-1251

[34] Lopez-Siles M, Duncan SH, Garcia-Gil LJ, Martinez-Medina M (2017) Faecalibacterium prausnitzii: from microbiology to diagnostics and prognostics. ISME J 11(4):841–852. 10.1038/ismej.2016.176 28045459 10.1038/ismej.2016.176 PMC5364359

[35] Jacob N, Jaiswal S, Maheshwari D et al (2020) Butyrate induced Tregs are capable of migration from the GALT to the pancreas to restore immunological tolerance during type-1 diabetes. Sci Rep 10(1):19120. 10.1038/s41598-020-76109-y 33154424 10.1038/s41598-020-76109-y PMC7644709

[36] Moon C, Baldridge MT, Wallace MA et al (2015) Vertically transmitted faecal IgA levels determine extra-chromosomal phenotypic variation. Nature 521(7550):90–93. 10.1038/nature14139 25686606 10.1038/nature14139 PMC4425643

[37] Paramsothy S, Kamm MA, Kaakoush NO et al (2017) Multidonor intensive faecal microbiota transplantation for active ulcerative colitis: a randomised placebo-controlled trial. Lancet 389(10075):1218–1228. 10.1016/S0140-6736(17)30182-4 28214091 10.1016/S0140-6736(17)30182-4

[38] Hansen IS, Baeten DLP, den Dunnen J (2019) The inflammatory function of human IgA. Cell Mol Life Sci 76(6):1041–1055. 10.1007/s00018-018-2976-8 30498997 10.1007/s00018-018-2976-8 PMC6513800

[39] Vishnu Prasoodanan PK, Sharma AK, Mahajan S et al (2021) Western and non-western gut microbiomes reveal new roles of Prevotella in carbohydrate metabolism and mouth-gut axis. NPJ Biofilms Microbiomes 7(1):77. 10.1038/s41522-021-00248-x 34620880 10.1038/s41522-021-00248-x PMC8497558

[40] Christensen L, Vuholm S, Roager HM et al (2019) Prevotella abundance predicts weight loss success in healthy, overweight adults consuming a whole-grain diet ad libitum: a post hoc analysis of a 6-wk randomized controlled trial. J Nutr 149(12):2174–2181. 10.1093/jn/nxz198 31504699 10.1093/jn/nxz198

