# Supplementary material for: Gut microbiome shifts in people with type 1 diabetes are associated with glycaemic control: an INNODIA study
Source: Diabetologia. 2024 Jun 4;67(9):1930–42. doi: 10.1007/s00125-024-06192-7 (PMC11410864; doi:10.1007/s00125-024-06192-7)
Supplement: Supplementary file 1 — ESM (PDF 74 KB) [file 125_2024_6192_MOESM1_ESM.pdf]

Electronic supplementary material for

Gut microbiome shifts in people with type 1 diabetes are associated with glycemic control – an INNODIA study

Members of the INNODIA and INNODIA HARVEST consortia

Consortium members

Mathieu	Chantal	KU Leuven, Belgium
Gillard	Pieter	KU Leuven, Belgium
Casteels	Kristina	KU Leuven, Belgium
Overbergh	Lutgart	KU Leuven, Belgium
Dunger	David	University of Cambridge, United Kingdom
Wallace	Chris	University of Cambridge, United Kingdom
Evans	Mark	University of Cambridge, United Kingdom
Thankamony	Ajay	University of Cambridge, United Kingdom
Hendriks	Emile	University of Cambridge, United Kingdom
Bruggraber	Sylvaine	University of Cambridge, United Kingdom
Marcoveccchio	Loredana	University of Cambridge, United Kingdom
Peakman	Mark	King's College London, United Kingdom
Tree	Timothy	King's College London, United Kingdom
Morgan	Noel G.	University of Exeter, United Kingdom
Richardson	Sarah	University of Exeter, United Kingdom
Todd	John A	University of Oxford, United Kingdom
Wicker	Linda	University of Oxford, United Kingdom
Mander	Adrian	Cardiff University, United Kingdom
Dayan	Colin	Cardiff University, United Kingdom
Alhadj Ali	Mohammad	Cardiff University, United Kingdom
Pieber	Thomas	Medical University of Graz, Austria
Eizirik	Decio L.	Universite Libre de Bruxelles, Belgium
Cnop	Miriam	Universite Libre de Bruxelles, Belgium
Brunak	Søren	University of Copenhagen, Denmark
Pociot	Flemming	Herlev University Hospital, Region Hovedstaden, Denmark
Johannesen	Jesper	Herlev University Hospital, Region Hovedstaden, Denmark
Rossing	Peter	Herlev University Hospital, Region Hovedstaden, Denmark
Legido Quigley	Cristina	Herlev University Hospital, Region Hovedstaden, Denmark
Mallone	Roberto	Cochin Institute Paris, France
Scharfmann	Raphael	Cochin Institute Paris, France
Boitard	Christian	Cochin Institute Paris, France
Knip	Mikael	University of Helsinki, Finland
Otonkoski	Timo	University of Helsinki, Finland
Veijola	Riitta	University of Oulu, Finland
Lahesmaa	Riitta	University of Turku, Finland
Oresic	Matej	University of Turku, Finland
Toppari	Jorma	University of Turku, Finland

Danne	Thomas	Children's and Youth Hospital Hannover, Germany
Ziegler	Anette G.	Helmholtz Zentrum Muenchen, Germany
Achenbach	Peter	Helmholtz Zentrum Muenchen, Germany
Rodriguez-Calvo	Teresa	Helmholtz Zentrum Muenchen, Germany
Solimena	Michele	TU Dresden, Germany
Bonifacio	Ezio E.	TU Dresden, Germany
Speier	Stephan	TU Dresden, Germany
Holl	Reinhard	University of Ulm, Germany
Dotta	Francesco	University of Siena, Italy
Chiarelli	Francesco	University of Chieti, Italy
Marchetti	Piero	University of Pisa, Italy
Bosi	Emanuele	University Vita-Salute San Raffaele, Italy
Cianfarani	Stefano	Bambino Gesù Children's Hospital, Italy
Ciampalini	Paolo	Bambino Gesù Children's Hospital, Italy
De Beaufort	Carine	Centre Hospitalier de Luxembourg, Luxemburg
Dahl-Jørgensen	Knut	Oslo University Hospital, Norway
Skrivarhaug	Torild	Oslo University Hospital, Norway
Joner	Geir	Oslo University Hospital, Norway
Krogvold	Lars	Oslo University Hospital, Norway
Jarosz-Chobot	Przemka	Medical University of Silesia, Poland
Battelino	Tadej	University of Ljubljana, Slovenia
Thorens	Bernard	University of Lausanne, Switzerland
Gotthardt	Martin	Radboud University Medical Center, The Netherlands
Roep	Bart O.	Leiden University Medical Center, The Netherlands
Nikolic	Tanja	Leiden University Medical Center, The Netherlands
Zaldumbide	Arnaud	Leiden University Medical Center, The Netherlands
Lernmark	Ake	Lund University, Sweden
Lundgren	Marcus	Lund University, Sweden
Costacalde	Guillaume	Univercell-Biosolutions, France
Strube	Thorsten	Sanofi, Germany
Schulte	Anke M.	Sanofi, Germany
Nitsche	Almut	Sanofi, Germany
Peakman	Mark	Sanofi, United States
Vela	Jose	Sanofi, United States
Von Herrath	Matthias	Novo Nordisk, Denmark
Wesley	Johnna	Novo Nordisk, Denmark
Napolitano-Rosen	Antonella	GlaxoSmithKline, United Kingdom
Thomas	Melissa	Eli Lilly, United Kingdom
Schloot	Nanette	Eli Lilly, United Kingdom
Goldfine	Allison	Novartis Pharma AG, Switzerland
Waldron-Lynch	Frank	Novartis Pharma AG, Switzerland
Kompa	Jill	Novartis Pharma AG, Switzerland
Vedala	Aruna	Novartis Pharma AG, Switzerland
Hartmann	Nicole	Novartis Pharma AG, Switzerland
Nicolas	Gwenaelle	Novartis Pharma AG, Switzerland
van Rampelbergh	Jean	Imcyse SA, Belgium
Bovy	Nicolas	Imcyse SA, Belgium
Dutta	Sanjoy	Juvenile Diabetes Research Foundation, United States
Soderberg	Jeannette	Juvenile Diabetes Research Foundation, United States
Ahmed	Simi	Juvenile Diabetes Research Foundation, United States
Martin	Frank	Juvenile Diabetes Research Foundation, United States
Latres	Esther	Juvenile Diabetes Research Foundation, United States

Agiostratidou	Gina	The Leona M. and Harry B. Helmsley Charitable Trust, United States
Koralova	Anne	The Leona M. and Harry B. Helmsley Charitable Trust, United States

2. Associated clinical sites

Willemsen	Ruben	Barts Health NHS Trust, United Kingdom
Smith	Anne	Northampton General Hospital NHS Trust, United Kingdom
Anand	Binu	West Suffolk NHS FT, United Kingdom
Datta	Vipan	Norfolk & Norwich University NHS FT, United Kingdom
Puthi	Vijith	North West Anglia NHS FT, United Kingdom
Zac-Varghese	Sagen	East & North Hertfordshire NHS Trust, United Kingdom
Dias	Renuka	Birmingham Women's and Children's NHS FT, United Kingdom
Sundaram	Premkumar	University Hospitals of Leicester NHS Trust, United Kingdom
Vaidya	Bijay	Royal Devon & Exeter NHS FT, United Kingdom
Patterson	Catherine	NHS Fife, United Kingdom
Owen	Katharine	Oxford University Hospitals NHS FT, United Kingdom
Dayan	Colin	Cardiff & Vale University Health Board, United Kingdom
Piel	Barbara	Queen Elizabeth Hospital, King's Lynn FT, United Kingdom
Heller	Simon	Sheffield Teaching Hospitals NHS FT, United Kingdom
Randell	Tabitha	Nottingham University Hospitals NHS Trust, United Kingdom
Gazis	Tasso	Nottingham University Hospitals NHS Trust, United Kingdom
Bismuth Reisman	Elise	Hospital Robert Debre, France
Carel	Jean-Claude	Hospital Robert Debre, France
Riveline	Jean-Pierre	Hospital Lariboisiere, France
Gautier	Jean-Francois	Hospital Lariboisiere, France
Andreelli	Fabrizio	Hospital Lapitie-Salpetriere, France
Travert	Florence	Hospital Bichat Claude Bernard, France
Cosson	Emmanuel	Hospital Jean-Verdier, France
Penfornis	Alfred	Centre Hospitalier Sud-Francilien, France
Petit	Catherine	Centre Hospitalier Sud-Francilien, France
Feve	Bruno	Hospital St Antoine, France
Lucidarme	Nadine	Hospital Jean-Verdier Pediatrie, France
Cosson	Emmanuel	Hospital Avicenne, France
Beressi	Jean-Paul	Hospital Andre Mignot, France
Ajzenman	Catherina	Hospital Andre Mignot Pediatrie, France
Radu	Alina	Hospital Europeen Georges-Pompidou, France
Greteau-Hamoumou	Stephanie	Hospital Louis Mourier, France
Bibal	Cecile	Hospital Kremlin Bicetre, France
Meissner	Thomas	Universitatsklinikum der Heinrich-Heine-Univeritat Dusseldorf, Germany
Heidtmann	Bettina	Katholisches Kinderkrankenhaus Wilhelmstift, Germany
Toni	Sonia	AOU Meyer, Italy
Rami-Merhar	Birgit	Medical University of Vienna, Austria
Eeckhout	Bart	Algemeen Ziekenhuis Geel Sint-Dimpna Geel, Belgium
Peene	Bernard	Algemeen Ziekenhuis Geel Sint-Dimpna Geel, Belgium
Vantongerloo	N	Algemeen Ziekenhuis Geel Sint-Dimpna Geel, Belgium
Maes	Toon	Imeldziekenhuis Bonheiden, Belgium
Gommers	Leen	Imeldziekenhuis Bonheiden, Belgium
